# A Retrospective, Observational Study to Assess the Efficacy, Safety, and Tolerability of Dietary Fiber Supplemental Combination in Overweight or Obese Patients

**DOI:** 10.7759/cureus.19973

**Published:** 2021-11-28

**Authors:** Ami Shah, Sabiha Siddiqui, Salome Benjamin, Mansi Patil, Himanshu P Tayade

**Affiliations:** 1 Dietetics, DietCastle Clinic, Mumbai, IND; 2 Department of Nutrition, Parsee General Hospital, Mumbai, IND; 3 Department of Nutrition, Jupiter Hospital, Thane, IND; 4 Department of Nutrition, Marian Cardiac Center, Pune, IND; 5 Pharmacology, Mediscience Healthcare Communications Private Limited, Navi Mumbai, IND

**Keywords:** bodyweight, dietary fiber, inulin, guar gum, obese, overweight, weight reduction, bmi, safe, effective

## Abstract

Study Objective

To assess the efficacy, safety, and tolerability of dietary fiber supplementation combination (DFSC) in decreasing the bodyweight (BW) and body mass index (BMI) of obese or overweight patients.

Methods

This was a retrospective, observational, multicentric, prescription event monitoring study. Forty-two overweight to obese individuals consumed DFSC, a combination of inulin, partially hydrolyzed guar gum, and resistant maltodextrin along with dietary and physical activity interventions. The cases had the following diet intake: 45%-55% carbohydrate, 15%-20% protein, and 20%-25% fat, with 15 g visible fat/day and 18-24 g DFSC daily for 12 consecutive weeks with vigorous diet monitoring fortnightly.

Results

The mean age of the patients was 40.74 years (standard deviation (SD): 12.16). The mean bodyweight and BMI of the patients were 80.63 kg (SD: 14.34) and 32.24 kg/m^2^ (SD: 13.98), respectively, at the baseline. At the end of weeks 4, 8, and 12, diet therapy and DFSC showed statistically significant reductions in the mean bodyweight and BMI as follows: 3.03 kg (SD: 01.24) and 1.18 kg/m^2^ (SD: 00.52) (*p* = 0.001), 5.70 kg (SD: 02.21) and 2.31 kg/m^2^ (SD: 01.08) (*p* = 0.001), and 7.82 kg (SD: 03.06) and 3.27 kg/m^2^ (SD: 01.86) (*p* = 0.001), respectively. Healthcare professionals rated diet therapy and DFSC as good to excellent for their efficacy and safety in 97.6% of the cases, and adverse event was not reported in any case with DFSC.

Conclusion

Dietary fiber supplemental combination with proper diet therapy/modification was found to be safe and effective in causing significant weight reduction in obese or overweight patients. However, a large multicentric study needs to be conducted.

## Introduction

Obesity is a global health concern. Obesity is associated with many diseases, viz., type 2 diabetes mellitus (T2DM), dyslipidemia, atherosclerosis and coronary heart disease, hypertension, and polycystic ovary syndrome (PCOS) [1,2].

It is well known that reduced dietary calorie intake and increased physical activities are useful in reducing bodyweight (BW). Reduced dietary calorie intake is achievable by regulating dietary portion size or reducing calorie-dense food in the diet with the maintenance of the amount of food [3].

There has been an inverse association between dietary fiber consumption and bodyweight [3]. The beneficial effect of the consumption of dietary fiber in obesity appears to be due to its assistance in controlling dietary calorie intake and appetite [4]. In India, there has been diversity in the intake of dietary fiber among different socioeconomic groups. The consumption of dietary fiber appears to be lesser in women [5].

This study was conducted to assess retrospectively the efficacy, safety, and tolerability of dietary fiber supplementation combination (DFSC) in decreasing the bodyweight (BW) and body mass index (BMI) of obese or overweight individuals. To our knowledge, this is the first-ever observational study in the world on the assessment of the efficacy and safety of dietary fiber supplementation of a combination of fibers such as inulin, partially hydrolyzed guar gum, and resistant maltodextrin.

## Materials and methods

Study objective

To assess the efficacy, safety, and tolerability of dietary fiber supplemental combination (DFSC) of inulin, partially hydrolyzed guar gum, and resistant maltodextrin in obese or overweight cases.

Study design

This was a retrospective, observational, multicentric, prescription event monitoring study. This study was conducted in accordance with the standard procedures/protocols of conducting retrospective studies [6]. Retrospective data of patients were collected between the period of August 2019 and January 2020. All ethical principles were followed while conducting this study.

Assessment of the test product

Forty-two overweight to obese individuals consumed dietary fiber supplementation along with dietary and physical activity interventions. The test medication (DFSC) (Fitofy, Nucgnex Lifesciences Private Limited, Pune, India) and its compositions are shown in Table 1.

**Table 1:** Composition of dietary fiber supplemental combination *No added sugar (natural sugar may come from other ingredients added)

Nutrients	Unit	Per 100 g	Per serving (6 g)
Energy	kcal	209	12.5
Protein	g	0	0
Fat	g	0.1	0.006
Total carbohydrate	g	92	5.52
Sugar (sucrose)*	g	0	0
Soluble dietary fiber	g	80	4.8

The following dosages of DFSC powder were followed: for obese or overweight patients, 18-24 g DFSC/day (mix one full scoop (6-8 g approximately) of DFSC powder in 250-300 mL water and consume thrice daily (15-20 minutes before meals (pre-lunch and pre-dinner) and 30 minutes pre-bedtime) for 12 consecutive weeks). Approximately 80% of patients consumed DFSC powder with water with no added sugar, and approximately 20% of patients consumed it with buttermilk with no added sugar.

Diet pattern

The following diet patterns before and during the study were followed by most of the patients.

Diet Pattern Before the Study

Most of the patients were on the following approximate macronutrient pattern of diet before therapy: 60%-70% carbohydrate, 8%-12% protein, and 25%-30% fat, with 30 g visible fat/day and less fiber intake.

Diet Pattern During the Study

Most of the patients were on the following approximate macronutrient pattern of diet during the diet therapy period: 45%-55% carbohydrate, 15%-20% protein, and 20%-25% fat, with 15 g visible fat/day.

Calories consumed were 1500-1800 kcal/day on weekdays and approximately 2000 kcal/day on weekends for 12 weeks in all patients. All patients were given standard advice regarding healthy food choices in their diet and physical activity. There was vigorous diet monitoring fortnightly for 12 weeks, and a reduction of 200 kcal/day consumption in the diet was decided on a case-to-case basis if needed by reviewing the calorie consumption by every patient.

No other nutrient supplementation or weight-reducing medicines were consumed by any patient during 12 weeks of fiber therapy.

Physical activity

All patients had undergone the following physical activity-related interventions: brisk walking for 30-45 minutes a day for three days in a week and either aerobic exercise, e.g., cardiac exercise/high-intensity exercise (an exercise strategy alternating short periods of anaerobic exercise with less-intense recovery periods until too exhausted to continue, e.g., push-ups, sit-ups, lunges, crunches, and running), or resistant training for 30-45 minutes a day for three days in a week during the study duration. The pattern of physical activity for all patients was similar before and during the study.

Data collection and follow-up

Observation of Cases

Forty-two overweight to obese individuals were considered for observation in this study at two centers. Data for each subject was documented in a prescription event monitoring form.

Study Visits

The following visits were considered to assess efficacy parameters: first visit, screening day/baseline visit (Day 0); second visit, at the end of four weeks (Day 28); third visit, at the end of eight weeks (Day 56); and fourth visit, at the end of 12 weeks (Day 84). As mentioned before, there was vigorous diet monitoring fortnightly for 12 weeks.

Subject inclusion criteria

Patients ≥17 years of age and both genders were considered. Patients who were overweight or obese were considered for assessment.

Efficacy parameters

The following parameters were assessed: bodyweight (BW) in kg and body mass index (BMI). BMI is calculated by dividing an individual’s weight in kilograms by the square of height in meters.

Safety assessment

Patients were evaluated for their safety based on patients’ opinions on the tolerability of the DFSC powder and healthcare professionals’ opinions on the safety of the DFSC powder.

Concomitant medication

If any concomitant medication was given to the patient, the same was recorded. Six, one, and two patients were on hypoglycemic, antihypertensive, and anti-hypercholesterolemic medications, respectively.

Statistical analysis

The statistical analysis of 42 individuals was carried out using the statistical software SPSS version 10.0. The descriptive analysis of demographics and the Student’s t-test of significance were used.

## Results

Demographic data

Demographic data are shown in Table 2. The mean age of the patients was 40.74 years, and the mean bodyweight was 80.63 kg (Table 2). Female cases were with a higher percentage.

**Table 2:** Demographic data SD: standard deviation

Parameters	N = 42
Age (years)	
Mean ± SD	40.74 ± 12.16
Range	18.00–82.00
Weight (kg)	
Mean ± SD	80.63 ± 14.34
Range	50.00–117.30
Gender (N (%))	
Male	15 (35.7)
Female	27 (64.3)

Efficacy evaluation

**Figure 1:**
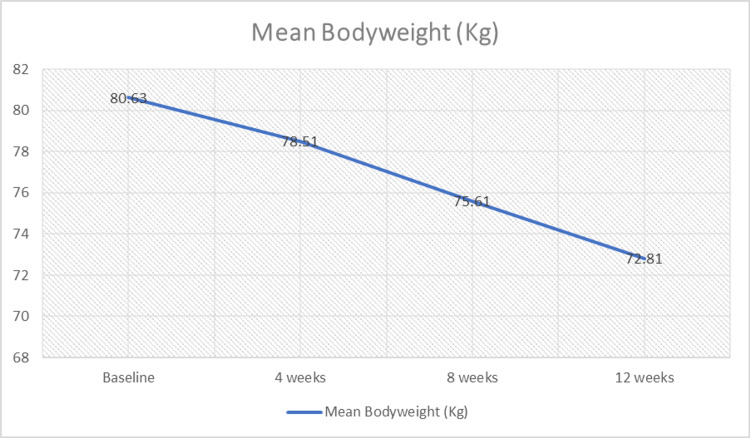
Changes in the mean bodyweight among the study cases with diet therapy and DFSC DFSC: dietary fiber supplemental combination

**Figure 2:**
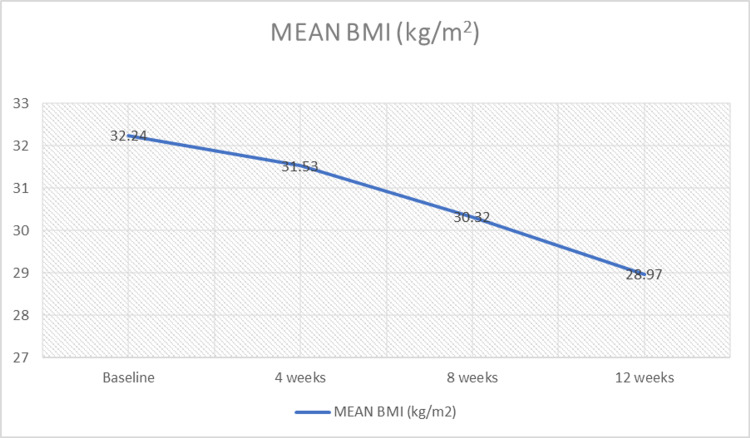
Changes in the mean body mass (BMI) among the study cases with diet therapy and DFSC DFSC: dietary fiber supplemental combination

The changes in bodyweight and BMI are shown in Table 3. At the end of weeks 4, 8, and 12, diet therapy and DFSC showed statistically significant reductions in the mean bodyweight and BMI as follows: 3.03 kg and 1.18 kg/m^2^, 5.70 kg and 2.31 kg/m^2^, and 7.82 kg and 3.27 kg/m^2^, respectively. The results are depicted in Figure 1 and Figure 2.

**Table 3:** Changes in the bodyweight and body mass (BMI) with diet therapy and DFSC Mean diff: mean difference (Student’s t-test); *: significant difference; SD: standard deviation; DFSC: dietary fiber supplemental combination

Duration in weeks	Mean bodyweight (kg) (mean ± SD) (N = 42)	Mean BMI (kg/m^2^) (mean ± SD) (N = 42)
Baseline	80.63 ± 14.34	32.24 ± 13.98
4	78.51 ± 13.67	31.53 ± 14.24
8	75.61 ± 13.57	30.32 ± 13.60
12	72.81 ± 13.25	28.97 ± 12.57
Mean diff (baseline – week 4) (p value)	-03.03 ± 01.24 (0.001)*	-01.18 ± 00.52 (0.001)*
Mean diff (baseline – week 8) (p value)	-05.70 ± 02.21 (0.001)*	-02.31 ± 01.08 (0.001)*
Mean diff (baseline – week 12) (p value)	-07.82 ± 03.06 (0.001)*	-03.27 ± 01.86 (0.001)*

Evaluation of tolerability, safety, and efficacy of DFSC by patients and healthcare professionals

Patients’ opinions on tolerability and efficacy are shown in Table 4. A view of good to excellent tolerability with DFSC was opined by 95.3% of the patients, and 100% of the patients opined that diet therapy along DFSC has good to excellent efficacy.

Healthcare professionals’ opinions on safety and efficacy are demonstrated in Table 5. As per the healthcare professionals, good to excellent safety with DFSC was observed in 97.6% of the cases, adverse event was not reported in any patient, and good to excellent efficacy with diet therapy and DFSC was observed in 97.6% of the patients.

**Table 4:** Patients’ opinions on tolerability and efficacy DFSC: dietary fiber supplemental combination

Patients’ opinions on DFSC tolerability		
Opinion	No. of cases (N = 42)	Percentage
Poor	-	-
Fair	02	4.7
Good	26	62
Excellent	14	33.3
Patients’ opinions on diet therapy and DFSC efficacy		
Opinion	No. of cases (N = 42)	Percentage
Poor	-	-
Fair	-	-
Good	28	66.6
Excellent	14	33.4

**Table 5:** Healthcare professionals’ opinions on safety and efficacy DFSC: dietary fiber supplemental combination

Healthcare professionals’ opinions on DFSC safety		
Opinion	No. of cases (N = 42)	Percentage
Poor	-	-
Fair	01	2.4
Good	07	16.6
Excellent	34	81
Healthcare professionals’ opinions on diet therapy and DFSC efficacy		
Opinion	No. of cases (N = 42)	Percentage
Poor	-	-
Fair	01	2.4
Good	34	81
Excellent	7	16.6

## Discussion

It is concluded in “The Finnish Diabetes Prevention Study” that long-term weight loss can be achieved with a high-fiber and low-fat diet as it was found to be greater in reducing bodyweight than that with a high-fat and low-fiber diet with results of approximately 3 kg versus 0.7 kg bodyweight reduction, respectively, in three years. Researchers also claimed that a low-fat, low-energy-density and high-fiber diet caused continued bodyweight reduction. The same study reported that the low-carbohydrate diet was more effective than the conventional low-fat and low-energy diet in causing significant short-term weight reduction. This might be due to the overall energy deficiency [3]. The weight-reducing effect observed in the current study is consistent with “The Finnish Diabetes Prevention Study”; although the patients in the present study consumed a low-fiber diet, it was compensated with the consumption of DFSC, and that may have contributed to significant bodyweight reduction.

In a six-month study conducted by Cicero et al. in hypertensive and overweight patients, oral consumption of guar gum 20 minutes before meals (nearly 10 g/day divided into three doses) resulted in significant improvement in the BMI, fasting plasma glucose (FPG) and insulin (FPI) levels, glycosylated hemoglobin (HbA1c), homeostasis model assessment (HOMA) index, low-density lipoprotein cholesterol (LDL-C), and apolipoprotein B (ApoB) [7]. The BMI-reducing effect was also observed in the present study with diet therapy and guar gum containing DFSC.

As per the review by Howarth et al., various studies demonstrate the following: increased consumption of soluble fiber causes increased after-meal satiety effect and reduces consequent hunger, increased fiber intake leads to a weight reduction of approximately 2 kg in about four months, and a reduction in energy intake and bodyweight takes place with natural high-fiber diet or with a dietary fiber supplement [7]. Various studies conducted by Howarth et al., Liu et al., and Ludwig et al. indicate that a higher intake of dietary fiber is associated with bodyweight reduction [8-10]. As per a review by Anderson et al., the recommended dietary fiber intakes for children and adults are 14 g/1000 kcal [11].

In our study, diet therapy and DFSC (containing soluble fiber) showed significant reductions in the mean bodyweight. The patients were not on any bodyweight-reducing medicines, and no patient needed such medicines during the consecutive 12 weeks of fiber therapy. Excellent tolerability and safety profile with DFSC were observed. These results with DFSC are very encouraging. However, a randomized controlled study is warranted to evaluate the efficacy of DFSC. The physical activity may not have contributed much to significant reductions in the mean bodyweight and BMI as the pattern of physical activity for all the patients was similar before and during the study. Calorie restriction was not strictly followed by all the patients before getting involved in the study, and calorie-restricted diet consumption was strictly monitored for all the patients during the study, which appeared to have contributed to significant reductions in the mean bodyweight. The weight-reducing effect appears to be superior to that observed in previously conducted fiber-related studies. A superior effect may be due to fortnightly vigorous diet monitoring for 12 weeks, including a reduction of 200 kcal/day consumption in the diet on a case-to-case basis.

The following could be the mechanisms behind the beneficial use of dietary fiber for reducing bodyweight. Preclinical studies have shown that the ingestion of inulin-type fructans can regulate bodyweight via the promotion of endogenous glucagon-like peptide-1 (GLP-1) in the gut. GLP-1 is the key controller of food intake by promoting satiety that consequently reduces the intake of food to cause a decrease in bodyweight and BMI [12]. As per the review by Howarth et al., a direct effect of dietary fiber on the gastric distension or delayed gastric emptying leads to the sensation of fullness; dietary fiber has an indirect effect by secreting intestinal hormones, e.g., incretins; fibers may cause reduced energy intake by reducing the absorption of fat and protein [8]. A study by Lyly et al. concluded that guar gum fiber-containing beverage showed a greater satiety effect and lower desire to eat than that with the beverage without fiber [4]. In a randomized controlled trial, Pol et al. reported a reduction in the appetite with the consumption of prebiotic dietary fiber (obtained from chicory root inulin) containing oligofructose granola bars as snack replacements in overweight and obese adults [13].

In the present study, the satiety effect of dietary fiber supplemental combination was not evaluated. Although few patients from the current study communicated that their appetite was reduced, it needs to be evaluated further.

The following additional benefits were found with dietary fiber in various studies. Liu et al. concluded that inulin appears to reduce LDL-C among patients suffering from overweight or obesity, metabolic abnormalities including dyslipidemia, and T2DM, whereas glucose control and HDL-C improvement seem to be reported in T2DM cases [14]. As per a study conducted by Dall’Alba et al., the supplementary partially hydrolyzed guar gum (PHGG) improved metabolic and cardiovascular profiles due to reduction in glycated Hb (HbA1c), waist circumference (WC), serum trans-fatty acids (FA), and 24-hour urinary albumin excretion (UAE) [15]. As per Blake et al.,depolymerized guar gum-supplemented wheat bread reduces plasma cholesterol concentration in patients with hypercholesterolemia [16]. They also concluded that resistant maltodextrin seems to improve the risk factors of metabolic syndrome by improving glucose and lipid metabolism and by reducing visceral fat [16]. However, most of the parameters from the abovementioned studies, excluding bodyweight and BMI, were not assessed in the present study.

The current study is the first-ever study on the assessment of the effect of DFSC of inulin, partially hydrolyzed guar gum, and resistant maltodextrin. We hypothesize that the contents of DFSC may be a synergistic combination of fibers to support bodyweight-reducing effect with diet therapy, which needs to be evaluated further.

Limitations of the study

The efficacy of DFSC was assessed for three months only, and a long-term assessment of the efficacy is needed. This was an observational, uncontrolled, retrospective, prescription event monitoring study. Other overweight- or obesity-associated parameters, viz., body fat area, waist circumference, satiety effect, blood glucose parameters, and lipid profile, were not assessed during the study. Diet and physical activity are possible confounding factors in evaluating the effect of individual dietary components/supplements such as fiber. A randomized, controlled clinical trial in a greater number of subjects with control of all the confounding factors is warranted to evaluate the efficacy of the dietary fiber supplementation combination and to confirm the synergistic effect of this combination.

## Conclusions

As per this observational, retrospective, prescription event monitoring study, the dietary fiber supplemental combination of inulin, partially hydrolyzed guar gum, and resistant maltodextrin (DFSC) with proper diet therapy/modification is found to be safe and effective in causing significant weight reduction in obese or overweight patients. However, a large multicentric, randomized controlled study in a wider range of patients and a larger number of patients including more investigational parameters for longer duration needs to be conducted with ruling out possible confound factors.
